# Bilateral Obstructive Uropathy From a Large Uterine Fibroid Causing Severe Renal Impairment: A Case Report

**DOI:** 10.1155/crog/5579471

**Published:** 2026-02-26

**Authors:** Felicia Woron, Jessie Jones, Evan Shreck, Amanda Ulrich

**Affiliations:** ^1^ School of Medicine, University of Connecticut, Farmington, Connecticut, USA, uconn.edu; ^2^ Division of Minimally Invasive Gynecologic Surgery, University of Connecticut Health Center, Farmington, Connecticut, USA, uchc.edu; ^3^ Tallwood Urology & Kidney Institute, Hartford HealthCare, Meriden, Connecticut, USA

## Abstract

Obstructive uropathy from uterine fibroids causing renal impairment has been reported in the literature but appears to be rare, with no definitive guidelines for management. We present a case of a 33‐year‐old Black female G0P0 with the incidental finding of a posterior cul‐de‐sac fullness found on routine physical examination. A follow‐up ultrasound revealed a large posterior fibroid measuring 19.5 cm. At her follow‐up visit, an in‐depth history revealed heavy menstrual bleeding and chronic urinary frequency. Subsequent evaluation revealed a creatinine level of 4.0 mg/dL, new‐onset hypertension, and severe bilateral hydronephrosis secondary to bilateral ureteral obstruction from the fibroid. Bilateral percutaneous nephrostomy tubes were placed for renal decompression prior to surgery with improvement in her creatinine to 2.9 mg/dL. The fibroid was successfully removed in its entirety via open abdominal myomectomy. Six weeks after surgery, her creatinine level decreased to 1.7 mg/dL but still remained above her baseline of 0.6 mg/dL, indicating some element of irreversible kidney damage. This case highlights the need for clinicians to maintain a degree of suspicion for obstructive uropathy in patients with large uterine fibroids, especially in patients with urinary symptoms and hypertension. This case also highlights the need for clinicians to identify and practice close surveillance for large uterine fibroids, given their potential to cause urinary tract obstruction that can progress to severe renal impairment.

## 1. Introduction

Obstructive uropathy from uterine fibroids can cause severe and potentially permanent renal impairment. This complication has been discussed in case reports, case series, and a few retrospective studies and literature reviews [1–10]. However, since patients typically do not receive renal imaging at the time of fibroid evaluation, there are few estimates of prevalence. In one retrospective study of 216 women with fibroids seen at a hospital in Jamaica over a 5‐year period, 14.35% had evidence of hydronephrosis; of those, 61.9% had normal renal function [1]. Only one patient presented with end‐stage renal failure (GFR < 15) due to obstructive uropathy from uterine fibroids [1]. A single‐center Nigerian study found that the prevalence of obstructive uropathy and nephropathy in patients presenting with symptomatic uterine fibroids (*n* = 114) was 22.8% and 10.5%, respectively; 4.4% had bilateral hydronephrosis [2]. Case reports also describe bilateral hydronephrosis or mild renal impairment, but very few report creatinine values above 2.5 mg/dL at the time of presentation or management [3–10]. Risk factors for obstructive uropathy may include large uterine volumes and leiomyoma location [1, 2, 5, 11]. Nearly all cases report resolution of symptoms and improvement of impaired renal function following myomectomy, hysterectomy, or uterine artery embolization (UAE) [3–10]. Only patients who delayed surgical management or had comorbid renal disease had ongoing renal insufficiency after management with myomectomy, hysterectomy, or UAE [8, 9].

We present a case of an incidental finding of a posterior uterine fibroid causing bilateral ureteral obstruction for an unknown length of time. The patient′s medical history was significant for iron deficiency anemia and hyperthyroidism. Her critical renal impairment was only discovered at the time of creatinine level evaluation prior to radiologic imaging. Upon further evaluation, she was also found to have new‐onset hypertension and severe bilateral hydronephrosis requiring percutaneous nephrostomy (PCN) tubes for renal decompression. The patient′s creatinine levels remained elevated six months postoperatively.

## 2. Case Presentation

A 33‐year‐old Black female G0P0 with past medical history significant for hyperthyroidism and iron deficiency anemia presented to our minimally invasive gynecologic surgery (MIGS) clinic for evaluation of an enlarged uterus found incidentally on routine well‐woman examination. She reported having regular but heavy menses lasting 3–5 days, with the need to change pads/tampons every 3–4 hours on the first day. She reported longstanding urinary frequency but otherwise denied pelvic pain, flank pain, dysuria, hematuria, gastrointestinal symptoms, or symptoms of anemia. She had no past abdominal surgeries and took no medications. Family history was significant for ovarian cancer in a great‐great grandmother. The patient′s BMI was 28 kg/m^2^ and vitals were remarkable for an elevated blood pressure of 164/110. Physical exam was notable for an enlarged, nonmobile uterus that extended up to 2 cm above her umbilicus. Pelvic exam revealed posterior fullness and extension of the uterus to the bilateral pelvic sidewalls with no uterine mobility. Her last hemoglobin level had been 9.7 g/dL. Transabdominal and transvaginal pelvic ultrasound, ordered to evaluate the physical exam findings, revealed an 11.1 × 14.6 × 23.5 cm anteverted uterus with an 11.1 × 8.7 × 9.8 cm uterine fibroid.

**Figure 1:**
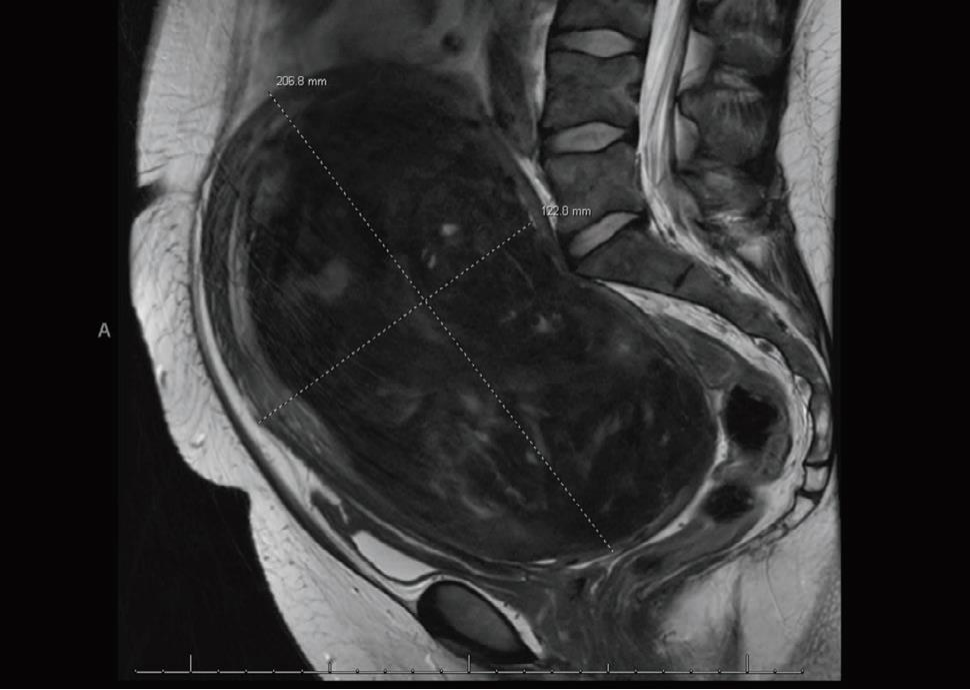
Pelvic MRI without contrast (sagittal view) showing dimensions of fibroid uterus with impingement at the pelvic brim.

**Figure 2:**
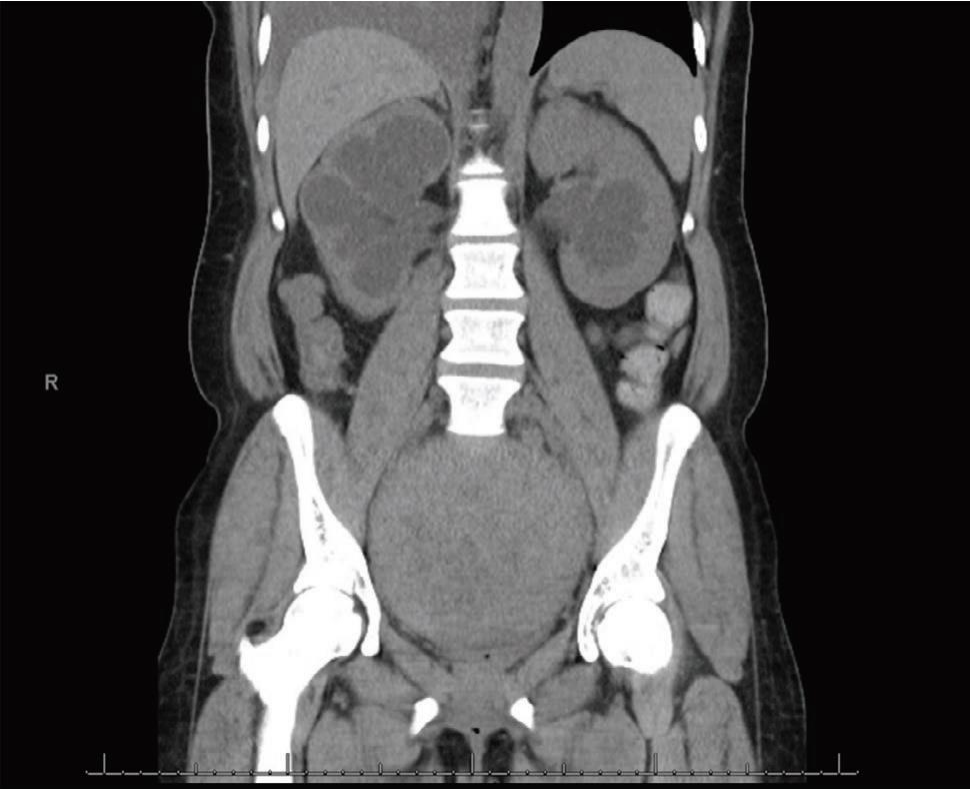
CT abdomen and pelvis without contrast (coronal view) showing severe bilateral hydronephrosis, right greater than left.

**Figure 3:**
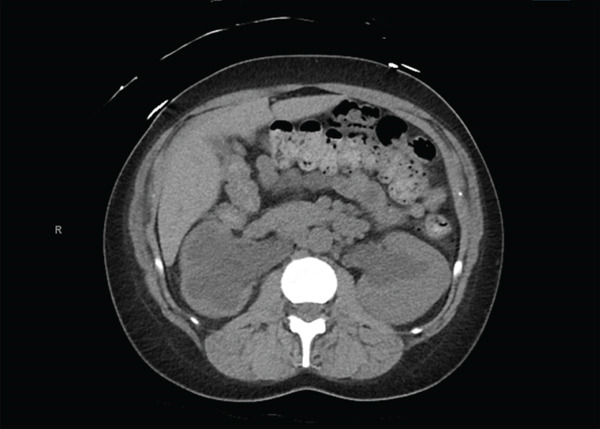
CT abdomen and pelvis without contrast (axial view) showing bilateral hydroureteronephrosis at the level of the ureteropelvic junction.

After extensive counseling, the patient desired minimally invasive, fertility‐sparing surgery via myomectomy. She was started on leuprolide with the goal of attempting to decrease the size of her fibroid and decrease intraoperative blood loss. Laboratory studies were ordered as well as a pelvic MRI for surgical planning. Her blood work revealed persistent iron deficiency anemia with a hemoglobin of 7.1 g/dL, and she subsequently began weekly IV iron infusions. In preparation for further imaging, the patient was found to have a creatinine level of 4.0 mg/dL. Chart review revealed that her baseline creatinine 6 years prior was 0.6 mg/dL, with a value of 1.10 mg/dL two years prior, which fell within normal limits. The patient reported no history of kidney disease and rare NSAID use. She had no symptoms of elevated creatinine. Her pelvic MRI revealed a single dominant intramural myoma in the posterior wall of the uterine corpus measuring 20 × 10 × 12 cm with extrinsic mass effect on the bladder and rectosigmoid colon, with resulting bilateral ureteral obstruction (Figure 1). Noncontrast CT revealed severe bilateral hydroureteronephrosis, right greater than left, with moderate cortical thinning of the right kidney indicating chronic obstruction and both ureters tapering at the level of the uterine myoma (Figures 2 and 3). The CT was performed to help delineate the level of ureteral obstruction, which was not clearly visualized on MRI. The kidneys had not been evaluated on initial pelvic ultrasound because there had not been significant clinical concern for renal pathology at that time. Upon receiving the imaging and renal function results, the patient was instructed to report to the emergency department immediately for evaluation.

In the emergency department, her creatinine was 3.6 mg/dL (eGFR 16) and blood pressure 165/116. She was admitted to the hospital for management of her renal insufficiency. Nephrology and urology were consulted. Initially, the rise in her creatinine was thought to be multifactorial with a concern for concurrent dehydration. When her creatinine failed to improve after IV hydration, the urology team recommended bilateral renal decompression. Due to the degree of obstruction and uterine size, bilateral PCN tubes were placed. Ureteral stents were not attempted due to a low probability of success given the degree of extrinsic ureteral compression from the fibroid. Per nephrology recommendation, she was closely monitored for three days with serial complete metabolic panels (CMPs). She was started on amlodipine for hypertension. Six days after admission, only mild improvement in her creatinine to 2.9 mg/dL was noted, and her renal function appeared to have plateaued. She was discharged with PCNs in place, though she experienced significant discomfort from them and failed multimodal analgesia. The patient strongly wished to proceed with surgery as soon as possible. In this context, abdominal myomectomy was planned for four days after her discharge.

**Figure 4:**
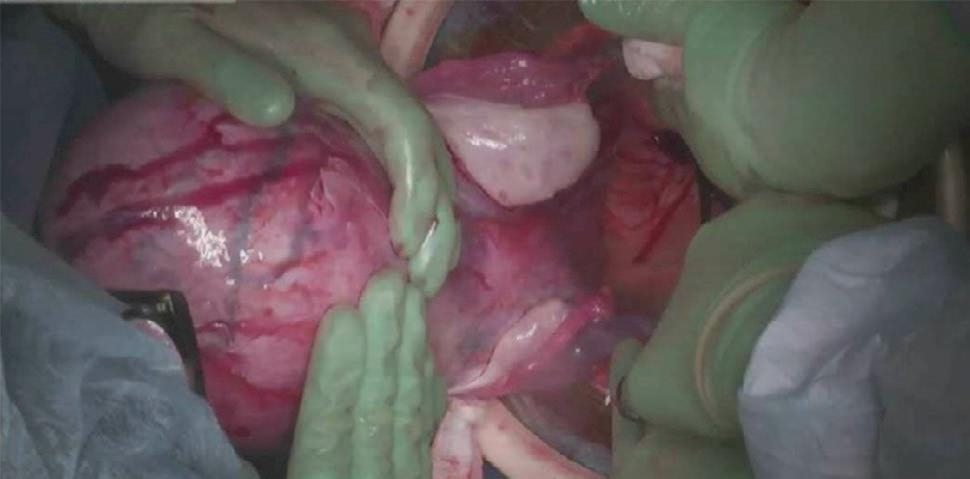
Enlarged uterus mobilized out of pelvis during myomectomy.

Prior to surgery, the patient was counseled on the necessity of performing the surgery via an open route due to the fibroid′s location and size. She was also counseled on the potential risk for hysterectomy. On the day of surgery, her creatinine level was 2.6 mg/dL. The myomectomy was performed via a midline vertical incision. Upon abdominal entry, her uterus measured 26 cm with the 20 cm posterior intramural fibroid filling the entire posterior cul‐de‐sac, compressing the bilateral ureters and the peritoneum overlying the sacral promontory. Myomectomy was performed in standard fashion, and the endometrial cavity was not entered. The myoma bed was closed in layers with v‐loc suture. The course of the ureters was identified and visualized away from the operative field at all times. Extracorporeally, the fibroid measured 19.5 × 13.0 × 11.5 cm and weighed 1676 g (Figure 4). Pathologic evaluation revealed a benign leiomyoma with focal degenerative changes. Her postoperative course was complicated by fever, atelectasis, and adynamic ileus, all of which resolved with conservative management by postoperative Day (POD) 4. The PCNs were removed on POD 5, following a successful clamping trial. On POD 1, her creatinine was 2.5 mg/dL. At the time of discharge on POD 5, her creatinine had improved to 2.0 mg/dL. At six weeks postoperatively, her creatinine had decreased to 1.7 mg/dL (eGFR 35). Renal and bladder ultrasound performed two months postoperatively showed complete resolution of the bilateral hydronephrosis, though cortical thinning of the right kidney remained. The patient has ongoing follow‐up with nephrology.

## 3. Discussion

Although obstructive uropathy from uterine fibroids appears to be rare, this case highlights the serious consequences that can occur in the absence of major symptoms.

There have been several published case reports and case series of patients with obstructive uropathy from uterine fibroids, some with concurrent renal impairment. Reported cases encompass patients aged 25–65 who presented with symptoms including severe vaginal bleeding or menorrhagia, urinary tract infection or pyelonephritis, and bulk symptoms [3–10]. There are also a few reported cases of patients presenting with new‐onset hypertension and its sequelae, including one report of an acute cerebral infarct [7, 9]. Although our patient did report heavy menstrual bleeding and urinary frequency, she was referred due to findings on routine well‐woman physical examination and did not seek care due to any particular symptom. At the time of evaluation, she was also recognized to have new‐onset hypertension, likely secondary to her renal dysfunction. This case therefore highlights the importance of noting new‐onset hypertension in the case of uterine fibroids and maintaining a degree of suspicion for obstructive uropathy as a possible cause.

Proposed risk factors for obstructive uropathy in the literature include uterine volumes larger than 200 cm^3^, posterior leiomyoma position, and incarceration of the leiomyoma beyond the pelvic brim (i.e., in the hollow of the sacrum) [1, 2, 5, 11]. Nearly all cases reported resolution of symptoms following myomectomy, hysterectomy, or UAE [1–11]. Our case suggests that imaging and evaluation of renal function should be considered in the setting of enlarged fibroid uterus or posterior uterine fibroids, or in cases where patients report concurrent urinary symptoms. Patients typically do not receive renal imaging at the time of fibroid evaluation, but based on fibroid size and location, renal imaging should be considered to identify obstructive uropathy and intervene before renal function abnormality.

Although several cases in the literature report concurrent impaired renal function, creatinine values generally ranged between 1.0–2.5 mg/dL, with only a few cases reported to have creatinine values above 2.5 mg/dL [4, 6, 7, 9, 10]. Our patient, who had a baseline creatinine of 0.6 mg/dL, was found to have a creatinine level of 4.0 mg/dL at the time of presentation. The duration of her obstruction is unknown. In hindsight, the patient reported that her abdomen had felt enlarged for at least five years.

Several reported cases required renal decompression prior to surgery, with ureteral stenting generally being attempted prior to PCN placement, and very few reports of patients requiring PCN placement [1, 3, 5–10]. Per recommendations from our urology colleagues and their suspicion that ureteral stents would not work due to extrinsic compression from the fibroid, our patient received bilateral PCNs. Specific guidelines for management do not currently exist. Even for other etiologies of obstructive uropathy, there is a consensus that urinary tract decompression is needed when the patient is septic or has abnormal renal function, but there is no consensus about the best method [12]. The choice between ureteral stents and PCNs appears to be based on clinical and institutional preference [12]. Ureteral stenting allows for greater patient comfort and independence and is generally attempted first, with PCNs typically reserved for cases of stent failure, malignant invasion of the bladder, or length of ureteral obstruction > 3 cm per a literature review [12].

The majority of published cases report complete resolution of hydronephrosis and recovery of renal function following definitive management in the form of hysterectomy, myomectomy, or UAE, and the subsequent removal of ureteral stents or PCNs if present [3–7, 10]. The few cases reporting persistent or worsening renal function encompassed patients of reproductive age who initially declined treatment due to concerns about surgery and future fertility, and a patient with baseline renal insufficiency [8, 9]. Our patient showed promising evidence of renal recovery after her myomectomy, with her creatinine decreasing from 2.9 mg/dL prior to surgery to 1.7 mg/dL six weeks after. Four months after myomectomy, her creatinine values were in the 1.4–1.7 mg/dL range. However, the long‐term course of her renal recovery is yet to be determined. Since there was not complete resolution of renal function, long‐term chronic kidney disease is suspected, presumably secondary to chronic ureteral obstruction.

Finally, it is important to note that Black women have consistently been shown to have a higher incidence, prevalence, and severity of uterine fibroids compared with their White counterparts, with age‐adjusted incidence rates estimated to be about three times greater [13, 14]. Black women are also more likely to experience symptomatic disease and diagnosis at an earlier age, leading to greater rates of surgical intervention [14]. Black women with uterine fibroids experience higher rates of surgical complications and hospital readmission [13]. Causes of this disparity likely include a complex interplay between genetic and environmental factors, with strong evidence for a contributing role from social determinants of health [13, 14]. Specific data about racial disparities in fibroids causing obstructive uropathy do not yet exist.

## 4. Conclusion

We highlight a rare case of bilateral obstructive uropathy from a uterine fibroid causing severe renal dysfunction. We point out the possibility that severe consequences can occur from mass effect, even in the absence of notable symptoms. We propose checking baseline kidney function when large pelvic masses are found, especially if concomitant new hypertension and urinary symptoms are present, to allow for quicker intervention. Additional large‐scale studies aimed at determining the prevalence of obstructive uropathy and renal impairment from uterine fibroids, as well as risk factors, may be helpful in determining appropriate screening guidelines and management for future cases. Nonetheless, this case demonstrates the necessity for close surveillance of large uterine fibroids and timely intervention when mass effect is present to avoid urinary tract obstruction that can progress to permanent renal impairment.

## Funding

No funding was received for this manuscript.

## Consent

Written consent has been obtained by this patient to publish this case report. A copy of the signed consent form is readily available for review.

## Conflicts of Interest

The authors declare no conflicts of interest.

## Data Availability

The data that support the findings of this study are available from the corresponding author upon reasonable request.
